# Anesthesia

**Published:** 1901-12

**Authors:** G. P. Haymore


### ANESTHESIA.*

BY G. P. HAYMORE, M.D.

It has long been recognized by the medical profession, that the most important—and it might be added the most dangerous—part of an operation, is the administration of the anesthetic. As every physician is called on at some time or other to administer either chloroform or ether, I want to mention some of the most important points in connection with—

(a) The choosing of an anesthetic.

(b) The administration and the treatment of accidents which sometimes occur during anesthesia.

(a) As a preliminary to every operation, where it is proposed to use a general anesthetic, the functional condition of the kidneys, lungs, heart, as well as the general condition of the patient, should receive careful attention ; and, while anesthetics are often administered without these precautions, yet it would be well to make it a "Golden Rule" never to give either chloroform or ether without making careful and repeated examinations of the urine, especially for albumen, casts and sugar, for by these means we may ofttimes be enabled to discover some latent disease of the renal structures, whereby the elimination of waste products is so lessened, that in

* Read before the Tri-State Medical Society of Alabama, Georgia and Tennessee.

many instances we may desist from an operation altogether, or until the functional activity of the kidney is vastly increased. In other cases of less degree, while in all probability chloroform is just so much irritating to the renal parenchyma as ether, yet the amount of the former necessary to produce and maintain complete anesthesia is so much less than the amount of the latter, it would be well in all cases to use chloroform.

The physiological action of both chloroform and ether on the respiratory and circulatory system is so well known—especially the direct depressing effect which chloroform has on the cardiac muscle—that it does not require special mention, but the lungs and heart should receive the same careful consideration as the urine, and in every case where he can discover any sign or symptom of cardiac disease, we should never administer chloroform, though we have Howard Kelley for authority, that chloroform is not contra-indicated in valvular diseases with good compensation.

If it should be noted during the examination that the arterial system of the patient has undergone calcareous or atheromatous degeneration, it would be well to remember that the vascular excitement incident to the stimulating effect of ether, might produce rupture of some small cerebral artery and apoplexy. Generally ether is considered to be safer than chloroform for anesthesia, though in point of fact when chloroform is used the amount needed is less, the time to produce anesthesia less, and subsequently there is less nausea and vomiting.

(3) The best method of administering chloroform is with the Esmarch inhaler; this should be placed close down over the mouth and nose of the patient, and the chloroform given regularly and slowly, drop by drop. This is a method which is opposed to the teachings of many on the subject, yet it is one which is advocated by Dr. Sayer, of New York, and one which I believe will gradually grow in favor. It takes less chloroform, less time to anesthetize the patient, and the excitement and struggle of the patient, which so often characterizes the primary stage of anesthesia, when the anesthetic is given so much diluted with air, will in a large majority of cases be avoided.

Ether is best given with Allis inhaler, and the method described by Allis will be found good.

The administration should be begun gradually; at first only a few drops given, and then as the patient begins to breathe more deeply and regularly the ether should be poured on in a small stream. By this method the patient will generally pass quietly under its influence in a few minutes. An operation is ofttimes hindered and harrassed throughout an operation by muscular movements on part of the patient, due to timidity of the anesthetizer in not administering enough of the anesthetic, and in order to avoid this we should be well acquainted with the signs of complete anesthesia. The most important are:

(1) Complete muscular relaxation associated with abolition of reflex movements.

(2) Deep snoring respiration.

(3) Conjunctival anesthesia.

It should be the effort of the anesthetizer to give the patient enough of the anesthetic, so that there will be just a faint reflex in the conjunctiva, and remember always any beyond this point of conjunctival anesthesia is dangerous.

It is very important that careful watch should be constant over the pulse and respitory movements, in order that any marked changes or signs of danger may be noted instautly. The most common symptoms of danger are:

(1) Failure of circulation, associated with a pallid or ashy expression of the features.

(2) Sudden and fixed dilation of the pupil.

(3) Shallow and rapid respiratory movements, or cessation of respiration.

(4) A cyanosed condition of the face, with protusion of eyeballs, due in majority of cases to obstruction of air entering the lungs, by the tongue falling back on the larynx.

If any of these first mentioned symptoms should occur, i. e. circulatory failure, respiratory failure, or sudden fixed dilation of the pupils, the anesthetizer should immediately lower the head of the patient and remove the mask in order to allow the patient more air, then if the condition does not speedily improve, proceed at once to measures of resuscitation.

In circulatory failure, to simply lower the head, remove the mask and give a hypodermic of strych, gr. 1-30, and nitro-glycerine, gr. 1-100, will in many cases be found effective.

Sometimes we have in a patient what is known as "respiratory forgetfulness." These are cases in which the patient ceases to breathe in the primary stage of anesthesia; to simply compress the sides of the chest will generally overcome this difficulty.

In graver instances of circulatory and respiratory collapse, resort will have to be had to artificial respiration. If an assistant is near, the best method is to have him elevate the body of the patient about 45°, then the anesthizer grasps the posterior portion of the chest, and by drawing it forward, produces inspiration and the reverse movement produces expiration.

This treatment in almost all cases will speedily restore the patient. If assistance cannot be had, then the Sylvester method should be tried. In cases in which the respiration is obstructed by the tongue falling back on the larynx, to simply extend the head of the patient and with the fingers push the angles of the jaws forward, will in a great many cases restore the respiratory movements.

After operation always remain with the patient until thoroughly aroused.

